# Supplementary material for: Nuclear spin noise tomography in three dimensions with iterative simultaneous algebraic reconstruction technique (SART) processing
Source: Magn Reson (Gott). 2020 Aug 6;1(2):165–73. doi: 10.5194/mr-1-165-2020 (PMC10500707; doi:10.5194/mr-1-165-2020)
Supplement: The supplement related to this article is available online at: https://doi.org/10.5194/mr-1-165-2020-supplement. [file mr-1-165-supplement.zip › supplementary_material.docx]

Coordinate system

Figure S1 shows the coordinate system used for describing the gradient directions and the data processing scheme in the main text.


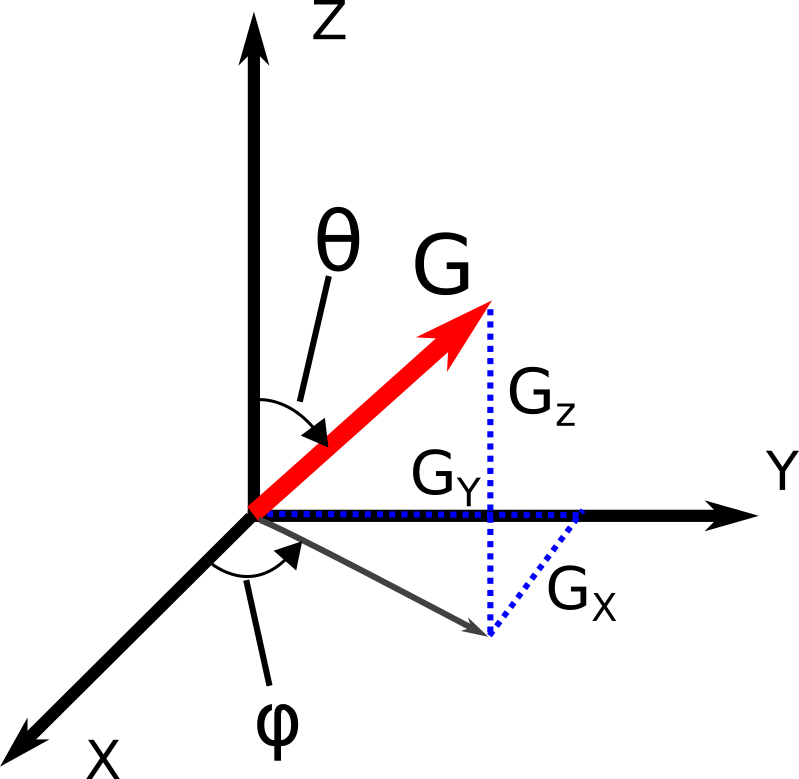


Figure S1: The standard three-dimensional cartesian coordinate system. The red arrow indicates an example gradient G to show the location of the angles. The blue dotted lines mark the cartesian coordinate vector components (G_x_, G_y_, G_z_) of the gradient G.
