# Supplementary material for: Nuclear spin noise tomography in three dimensions with iterative simultaneous algebraic reconstruction technique (SART) processing
Source: Magn Reson (Gott). 2020 Aug 6;1(2):165–73. doi: 10.5194/mr-1-165-2020 (PMC10500707; doi:10.5194/mr-1-165-2020)
Supplement: The supplement related to this article is available online at: https://doi.org/10.5194/mr-1-165-2020-supplement. [file mr-1-165-supplement.zip › mr-1-165-2020-supplement-title-page.pdf]

Supplement of Magn. Reson., 1, 165–173, 2020
https://doi.org/10.5194/mr-1-165-2020-supplement
© Author(s) 2020. This work is distributed under
the Creative Commons Attribution 4.0 License.

Open Access
MAGNETIC RESONANCE

Supplement of

Nuclear spin noise tomography in three dimensions with iterative simultaneous algebraic reconstruction technique (SART) processing

Stephan J. Ginthör et al.

Correspondence to: Norbert Müller (norbert.mueller@jku.at)

- mr-1-165-2020-supplement-title-page.pdf
- preprocess.py
- reconstruct.py
- supplementary_material.docx

The copyright of individual parts of the supplement might differ from the CC BY 4.0 License.
